# In silico prediction of pathogen's pandemic potential using the viral trait assessment for pandemics (ViTAP) model

**DOI:** 10.1093/pnasnexus/pgae558

**Published:** 2024-12-10

**Authors:** Charles H Jones, Marie Beitelshees, B Adam Williams, Andrew B Hill, Verna L Welch, Jane M True

**Affiliations:** Pfizer, 66 Hudson Boulevard, New York, NY 10018, USA; Bulmore Consulting, Lockport, NY 14094, USA; Pfizer, 66 Hudson Boulevard, New York, NY 10018, USA; Bulmore Consulting, Lockport, NY 14094, USA; Pfizer, 66 Hudson Boulevard, New York, NY 10018, USA; Pfizer, 66 Hudson Boulevard, New York, NY 10018, USA

## Abstract

Our world is ever evolving and interconnected, creating constant opportunities for disease outbreaks and pandemics to occur, making pandemic preparedness and pathogen management crucial for global health security. Early pathogen identification and intervention play a key role in mitigating the impacts of disease outbreaks. In this perspective, we present the Viral Trait Assessment for Pandemics (ViTAP) model to aid in the early identification of high-risk viruses that have pandemic potential, which incorporates lessons from past pandemics, including which key viral characteristics are important such as genetic makeup, transmission modes, mutation rates, and symptom severity. This model serves as the foundation for the development of powerful, quantitative tools for the early prediction of pandemic pathogens. The use of such a tool, in conjunction with other pandemic preparedness measures, can allow for early intervention and containment of the virus. This proactive approach could enable timely interventions, guiding public health responses, and resource allocation to prevent widespread outbreaks and mitigate the impact of emerging pathogens.

**Significance Statement**

The Viral Trait Assessment for Pandemics model introduces a novel risk assessment framework designed to facilitate the early identification of high-risk viruses with significant global impact. Incorporating insights from past pandemics, the model evaluates key viral characteristics, including genetic makeup, transmission modes, mutation rates, and symptom severity. This approach offers a predictive and proactive strategy to enhance pandemic preparedness and mitigate potential threats.

## Introduction

**Fig. 1.**
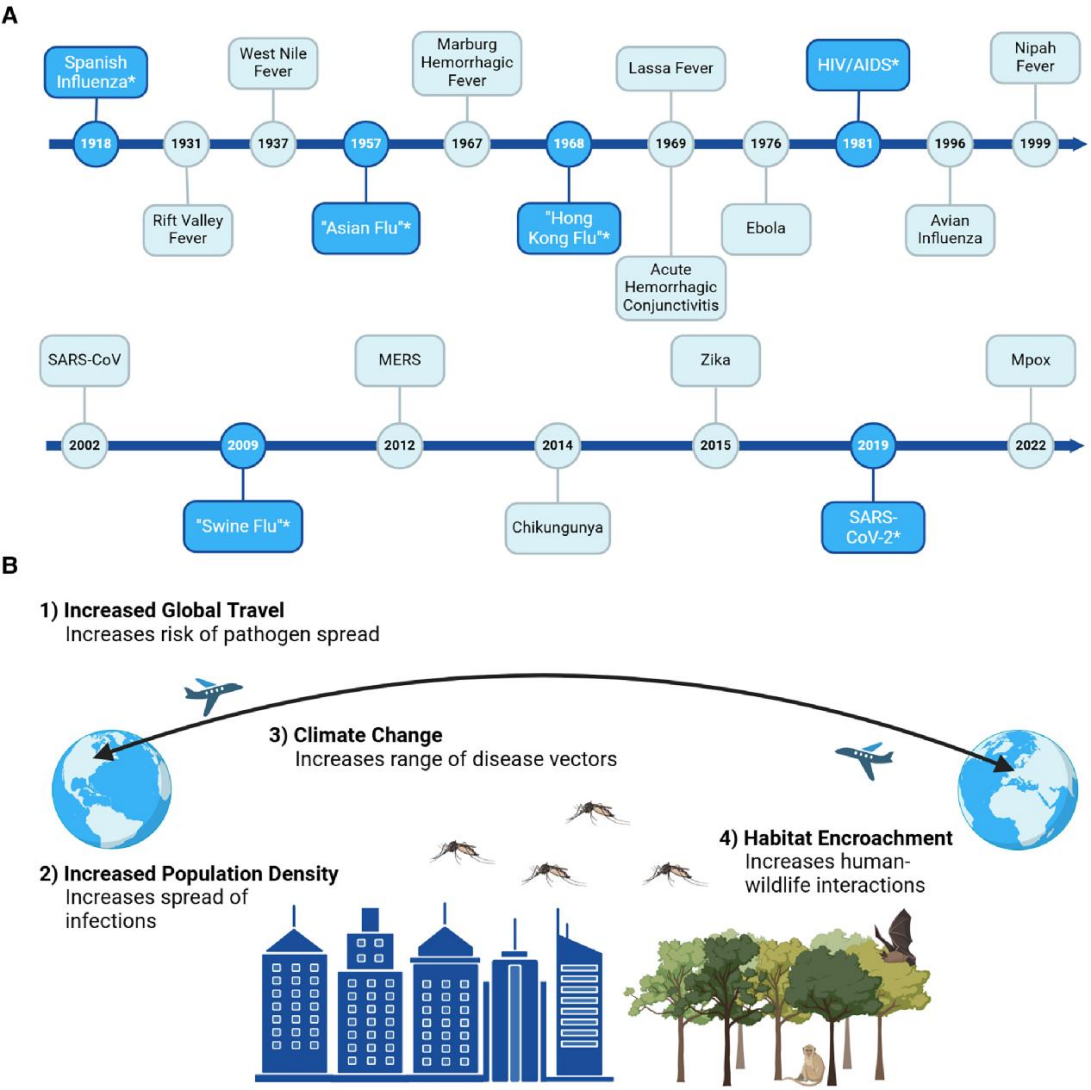
Past, present, and future of pandemic outbreaks. A) Outbreaks and pandemics from 1918 to present. Pandemics are designated with an asterisk (*) (1, 2). Created with BioRender.com. B) Factors increasing the risk of pandemic outbreaks. Created with BioRender.com.

Throughout history, humans have been plagued by pandemics (Figure 1A). From the Antoine Plague in 165 AD, which claimed the lives of up to a fifth of the Roman Empire's population (3), to the Spanish Flu of 1918, these widespread diseases have caused significant loss and disruption. A pandemic occurs when an infectious disease affects a large number of people across countries and continents, spreading rapidly due to human-to-human transmission (4). However, to be considered novel, a pandemic must involve a pathogen that may include a completely new virus or novel viral strain, resulting in high susceptibility and lack of immunity (4). The most recent novel pandemic, caused by the SARS-CoV-2 virus, has swept across the globe, causing over 770 million cases and approximately 7 million deaths as of October 2023 (5). This pandemic has had a profound impact on many aspects of our modern lives and has had a detrimental impact on the global economy through factors such as supply chain shortages and control measures like lockdowns (6).

Many think of pandemics like COVID-19 as a once-in-a-lifetime occurrence, and in fact there is approximately a 17% chance of experiencing a similar event in one's lifetime (7, 8). This number has been increasing over time and is expected to continue rising due to factors like global travel, climate change, and human encroachment on natural animal habitats (Figure 1B) (9). As such, it is more important than ever to rapidly identify viruses with high pandemic potential and introduce measures to limit their spread.

Early identification of high-risk viruses and the timely declaration of pandemics is key to mitigating their impact on a global population. The official declaration of a pandemic triggers coordinated international efforts, allowing for the mobilization of resources and the implementation of widespread public health measures. This includes global collaboration on vaccine development, information sharing, and the enforcement of travel and trade regulations to curb the spread of the virus. However, the World Health Organization's (WHO) three-month delay in declaring COVID-19 a pandemic, despite knowing in January that it was spreading globally, hindered the prompt implementation of containment measures, potentially allowing the virus to spread more extensively and rapidly (10). In such situations, risk prediction tools could provide counties, organizations, and companies guidance on when to initiate pandemic preparedness measures and how best to direct their resources.

Tools for early prediction of pandemic risk are currently limited, and the majority of tools that do exist are designed for influenza and are not designed for pandemic risk prediction before or early in an outbreak. The US Centers for Disease Control and Prevention's (CDC's) Influenza Risk Assessment Tool (IRAT) (11) and the WHO's Tool for Influenza Pandemic Risk Assessment (TIPRA) (12) are both tools designed to identify influenza A strains in animals with high pandemic potential. They consider viral characteristics like receptor binding, genomic characteristics, transmission in animal models, and antiviral treatment options, as well as factors such as population immunity, disease severity and pathogenesis, and antigenic relatedness in the target population. Risk factors related to ecology and epidemiology are also considered, such as global distribution in animals, infections in animals, and human infections. While factors used in these assessments are applicable to all viruses, the criteria were developed specifically for influenza, which may limit their use for other viruses.

The CDC offers a Pandemic Severity Assessment Framework to evaluate the potential impact of an influenza A pandemic based on its clinical severity and transmissibility (13). However, this model assumes a pandemic is already occurring and incorporates information that may not be available at the beginning or immediately after an outbreak, such as viral attachment rates, the basic reproduction number *R*_0_, case-fatality rates, and hospitalization rates. Determining *R*_0_ early in the COVID-19 pandemic, for example, was a significant challenge characterized by inconsistent estimates. This has been attributed to a lack of peer review and speed of information release driven by the urgency early in the pandemic, resulting in *R*_0_ values ranging from 1.4 to 6.49 (14, 15). Such inconsistencies demonstrate the degree of uncertainty involved in the initial stages of the pandemic.

Recognizing the need for a more comprehensive and adaptable approach, the Viral Trait Assessment for Pandemics (ViTAP) model has been developed to address the limitations of existing tools. This model evaluates a broad spectrum of viral characteristics, including genetic makeup, modes of transmission, mutation rates, and symptom severity, to assess the pandemic potential of various viruses beyond influenza. By incorporating learnings from past pandemics, the ViTAP model aims to provide a framework for the early identification of high-risk viruses. This proactive approach could enable timely interventions, thereby guiding public health responses and resource allocation to prevent widespread outbreaks and mitigate the impact of emerging pathogens.

## Getting into the genes of past pandemics

Analyzing a virus's genetic code provides insights into its ability to replicate, mutate, infect, and evade host immune response. While the expression of this genetic information depends on a complex interaction between environmental factors and host immunity, the presence of certain genes or mutations can serve as a powerful determinant of pandemic potential. By studying the genetic traits of pathogens that have caused past pandemics, such as smallpox, influenza A, HIV/AIDS, and SARS-CoV-2, insight can be gained that informs and guides future research and development in pandemic preparedness.

### Ability to mutate and adapt

The majority of modern pandemics have been driven by betacoronaviruses and influenza A (16). While betacoronavirus and influenza A viruses commonly circulate in the global population, causing the common cold, and seasonal flu, respectively, it is their ability to mutate rapidly, giving rise to novel strains that can overcome preexisting immunity, which makes these viruses particularly successful at reaching pandemic levels. Since the beginning of the 19^th^ century, there have been four novel influenza A pandemics: (i) the Spanish Flu (1918–1919), (ii) the Asian flu (1957–1958), (iii) the Hong Kong flu (1968–1969), and (iv) the H1N1 swine flu (2009–2010). There has also been one betacoronavirus pandemic, SARS-CoV-2, and two epidemics SARS-CoV, MERS-CoV.

Both betacoronaviruses and influenza A fall under the categorization of single-stranded RNA (ssRNA) viruses, which are highly susceptible to rapid mutation and adaptation (Table 1) (24). This category also includes another pandemic virus, HIV-1, whose high mutation rate has made the development of interventions against it particularly challenging. The impact of these pandemic viruses suggests that ssRNA viruses with high mutation rates have higher pandemic potential than non-ssRNA viruses. Determining the mutation rate of a virus involves observing changes in its genetic material over time, meaning that this information may not be readily available at the beginning of an outbreak. Instead, insights can be gained into the mutation rate of a novel virus by considering factors such as the genome's size and the replication machinery employed.

**Table 1.** Genomic characteristics of pandemic viruses.

Virus	Family/Genus	Nucleic acid Config.	Genome size (kp)	Replication machinery	Mutation Rate (Subs./site/year)	REF
Smallpox	Poxviridae/Orthopoxvirus	dsDNA	186	DNA polymerase errors	2 × 10^−6a^	(17, 18)
Influenza A	Orthomyxoviridae/Influenza A	ssRNA (−)	13–15	RNA polymerase errors	2 × 10^−3^	(19, 20)
HIV-1	Retroviridae/Lentivirus	ssRNA (+)	9.7	Error-prone reverse transcriptase, recombination	4.4 × 10^−3^	(19, 21, 22)
SARS-CoV-2	Coronaviridae/Betacoronavirus	ssRNA (+)	29.9	RNA polymerase errors, proofreading exonuclease	1 × 10^−3^ to 2 × 10^−3^	(23)

(−), Negative stranded; (+), Positive stranded; Config., Configuration; dsDNA, Double-stranded DNA; ssRNA, Single-Stranded RNA; Subs., Substitutions.

^a^The mutation rate of smallpox is not as well characterized as that of some other viruses, but estimates from various studies suggest a rate of approximately 2 × 10^−6^ substitutions per site per year. This estimate is based on the analysis of the smallpox virus genome and its comparison to other related orthopox viruses.

In many cases, there appears to be an inverse relationship between genome size and mutation rate (25). Small genomes are believed to be able to tolerate higher mutation rates as a larger proportion of mutation-free genomes are generated in each round of replication due to their small size (26). This trend is seen across the range of pandemic viruses examined in this perspective. HIV-1, for example, has the smallest genome size of 9.7 kb, and highest mutation rate among pandemic viruses and any other known biological entity (21). The DNA virus smallpox, in contrast, possesses the largest genome size and lowest mutation rate among the viruses evaluated. The correlation between genome size and mutation rate is strongly influenced by the type of genetic material (DNA vs RNA) (27). However, this inverse relationship is observed even among RNA viruses like betacoronaviruses and influenza A. SARS-CoV-2, for instance, has a large genome for an RNA virus and is nearly twice the size of that of an Influenza A virus, which, in turn, has a higher mutation rate.

The type of replication machinery also provides valuable insights into an RNA virus's mutation rate. Influenza A, for example, exhibits a high mutation rate due to its error-prone RNA polymerase, resulting in antigenic drift that necessitates annual vaccine updates (28–30). HIV has a much higher mutation rate than influenza A, driven by its error-prone reverse transcriptase enzyme which has allowed it to evade host immune responses and adapt to therapy (31). In contrast, coronaviruses like SARS-CoV, MERS-CoV, and SARS-CoV-2 encode a specialized RNA-dependent RNA polymerase with proofreading activity, which helps reduce the number of mutations resulting from incorrect nucleotide addition or misincorporation (32). This reduces the rate at which coronaviruses can generate mutations. Despite this, SARS-CoV-2 has demonstrated its ability to undergo significant genetic changes and mutations, resulting in new variants with heightened transmissibility, such as the Delta and Omicron strains (33). Factors contributing to this include its expansive genome, rapid replication rate, and the potential for recombination events among SARS-related coronaviruses (34).

Unlike RNA viruses, DNA viruses like smallpox exhibit significantly lower mutation rates due to the higher fidelity of their polymerases in charge of reproduction (18, 24). This results in reduced genetic diversity compared with RNA viruses, ultimately facilitating the development and effectiveness of vaccines and treatments against them. In the context of smallpox, this decreased genetic diversity played a crucial role in the successful development and wide implementation of the vaccine, leading to the complete eradication of the disease in the 1980s (35).

Viruses with segmented genomes possess another evolutionary mechanism known as genetic reassortment, a process where genetic material from different strains of the virus can be recombined during coinfection to create a new strain. This process is often attributed to a virus's ability to jump from animal to human hosts (36). Among the pandemic viruses assessed in this perspective, only influenza A has a segmented genome, with its eight RNA segments. A careful examination of past influenza pandemics reveals that reassortment between human and animal strains, leading to antigenic shift, created strains to which the contemporary population had little-to-no preexisting immunity (30). One example is the reassortment event in 2009, involving avian, human, and swine influenza viruses, which resulted in the emergence of a novel strain of H1N1. This strain infected 60 million to 184 million people worldwide and caused the loss of between 151,000 and 575,000 lives (37, 38).

### Genetic virulence and transmissibility factors

Once a virus with pandemic potential is discovered, the sequencing of its viral genome and antigenic characterization needs to take place rapidly. This process provides researchers with invaluable insights, such as whether the virus carries mutations which may increase its virulence or transmission rate. Having such knowledge at an early stage of the outbreak can enable researchers to assess risk and better prepare for, and manage, the potential pandemic.

Through the examination of pandemic viruses and their nonpandemic counterparts, it is clear that mutations in certain genes have significantly bolstered the transmissibility and virulence, ultimately resulting in epidemics that may become pandemics (Table 2). Variola major, the primary causative agent behind smallpox, has historically been one of the most destructive viruses in history, far exceeding the virulence of its counterpart, Variola minor. This can be attributed to genetic disparities in proteins governing the modulation of host immune response, such as those involved in cell tropism and cytopathic effects (e.g. B19, EEV, and SV40) (39). Similarly, HIV-1 and HIV-2, while both capable of causing AIDS, exhibit vastly different genetic profiles, leading to divergent epidemiological patterns. The absence of the Vpx gene in HIV-1, a gene present in HIV-2, accounts for the slower immune system degradation and less aggressive disease progression observed in the latter virus (43, 44). The envelope glycoprotein gp120, which facilitates the attachment of HIV-1 to CD4 receptors, has a significantly higher affinity in HIV-1 compared with HIV-2, contributing to the heightened transmissibility of HIV-1 (44). These genetic differences greatly influence the ways in which the viruses spread and cause disease, ultimately explaining why HIV-1 has become pandemic while HIV-2 remains confined geographically (47).

**Table 2.** Unique genetic characteristics of novel pandemic viruses.

Virus	Unique Genetic characteristics	Role of Virulence and transmissibility factors	REF
Smallpox (Variola Major) 12,000 BC–1980	Differences in D7L, EL3, and SPI-2	E3L: Inhibition of host PKR, DL7: Suppression of host IL-18, SPI-2: Suppression of host interferon response	(39)
1918 Influenza (H1N1) 1981–1919	Multiple changes in the receptor binding site of HA, unique mutations in NA, presence of PB1-F2, and a potent NS1	HA: Receptor binding and fusion, NS1: Inhibition of host interferon response NA: Viral release PB1-F2: Apoptosis of immune cells	(40)
1957 Influenza (H2N2) 1957–1958	HA, NA, and PB1 genes derived from an avian strain	HA: Receptor binding and fusion, NA: Viral release PB1: Viral replication and transcription	(41, 42)
1968 Influenza (H3N2) 1968–1969	HA and PB1 genes came from an avian influenza virus	HA: Receptor binding and fusion, PB1: Viral replication and transcription	(41, 42)
2009 Influenza (H1N1)pdm09 2009–2010	Reassortment involving swine, avian, and human strains; several changes in HA, unique mutations in NA and NS1, truncated PB1-F2	HA: Receptor binding and fusion, NA: Viral release NS1: Inhibition of host interferon response	(37, 41)
HIV-1 1980s–Present	Presence of Vrx gene and differences in the env gene encoding for the envelope glycoprotein gp120	Vpx: viral replication env: cellular entry	(43, 44)
SARS-CoV-2 2019–Present	Distinct furin cleavage site in the S protein, enhancing cell entry and transmission; ORF8 gene with potential roles in immune evasion and host adaptation	ORF8: Immune evasion, S protein: Receptor binding (ACE2) and fusion	(45, 46)

ACE2, Angiotensin-Converting Enzyme 2; env, Envelope; F2, Factor 2; gp120, Glycoprotein 120; HA, Hemagglutinin; HIV, Human immunodeficiency viruses; IL-18, Interleukin 18; NA, Neuraminidase; ORF8, Open Reading Frame 8; PB1, Polymerase Basic Protein 1; PB1-F2, Polymerase Basic Protein 1, Frame 2; PKR, Protein Kinase R; S, Spike; SPI-2, Serine Protease Inhibitor 2; Vpx, Viral accessory protein.

With the notable exception of the 1977 H1N1 influenza pandemic, which has been excluded from detailed analysis in this review as it was nearly identical to H1N1 strains circulating in the 1950s (48), pandemic strains of influenza are typically characterized by the emergence of a new hemagglutinin (HA) subtype that efficiently infects and transmits between humans (49, 50). This often results from reassortment events between different influenza viruses, leading to the creation of a novel strain to which the human population has limited immunity. While all four pandemics assessed in this perspective (1918 H1N1, 1957 H2N2, 1968 H3N2, and 2009 H1N1) are believed to have started with genetic reassortment events between human and avian viruses, the mutations responsible for increased virulence and transmissibility differed among these strains (41, 51). In the case of the 1918 H1N1 strain, distinct mutations in the HA and neuraminidase (NA) genes were identified as facilitating transmission among humans, while its NS1 protein was found to inhibit host immune responses (40). The 1957 H2N2 strain had avian-derived HA and PB1 genes, which likely contributed to its unique antigenicity (41, 42). In the 1968 H3N2 strain, avian-derived HA and PB1 genes were also detected, and mutations in the NA gene were believed to enhance its virulence (41, 42). Lastly, the 2009 H1N1 pandemic strain was the result of a reassortment event involving swine, avian, and human viruses, with mutations in the HA gene believed to be responsible for its ability to transmit between humans (37, 41). In all four pandemics, specific mutations in NS1 genes were thought to contribute to increased virulence by inhibiting the host immune responses (41).

Examining different virus species within the same family can offer valuable insights into mutations that enhance pandemic potential. SARS-CoV-2 exhibits genetic disparities compared to nonpandemic strains. Both SARS-CoV and SARS-CoV-2 infect cells through the ACE2 receptor, which is responsible for an enhanced ability to enter cells, evade the immune system, and adapt to humans (52). However, the ACE2 receptor of SARS-CoV-2 also possesses a unique furin cleavage site, resulting in higher affinity for the human ACE2 receptor compared with other viruses within the same family (53). This increased affinity is believed to contribute to the virus's increased transmissibility (54).

Assessing mutations in genetic virulence and transmissibility factors is key to evaluating the pandemic potential of a virus. It requires subject matter expertise to thoroughly examine the full scope and effects of identified mutations accurately. In vitro experiments may also be necessary to assess the impact of these mutations, enabling a comprehensive understanding of their implications. By leveraging scientific knowledge and analysis in a risk assessment model, insight can be gained into the pandemic potential of a virus.

## Interpreting the infection pathways of pandemic past

### Transmissibility

The transmissibility of infectious agents is one of the most important factors in determining the potential for a pandemic, as it is responsible for how efficiently a virus can spread from one person to another. Existing tools for assessing pandemic severity consider factors like attack rates and the basic reproduction rate (R_0_) (13). However, obtaining this information often requires time-consuming contact tracing efforts. Instead, at the start of an outbreak, this perspective proposes assessing viral characteristics impacting transmissibility such as genetic factors (described above) and modes of transmission.

Understanding the modes of transmission of pandemic-causing viruses is pivotal to assessing the risks associated with a specific virus. Respiratory droplets serve as the primary means of transmission for many pandemic viruses, including smallpox, influenza A, and SARS-CoV-2 (55–57). These minute particles are expelled into the air when an infected individual coughs or sneezes, can remain suspended for a considerable period, and are capable of traveling long distances (58). As a result, they become highly efficient agents for spreading infections. Moreover, secondary methods like airborne transmission (e.g. aerosols) and fomite transmission also contribute to the dissemination of pandemic viruses. While less effective modes of transmission, such as direct contact with bodily fluids and blood transfusions, are observed in cases like HIV (59), the presence of asymptomatic carriers, elevated viral loads, and various other factors significantly amplify the spread in such instances. Interestingly, despite the prevalence of vector-borne diseases, none have resulted in modern pandemics (60). Outbreaks like the Zika virus and Dengue have largely been limited by specific environmental requirements and dependency on a biological vector, which have effectively prevented them from reaching pandemic levels. Climate change, however, has the potential to significantly alter this dynamic. As the world continues to experience global warming, it is likely that the range of disease-carrying vectors, such as mosquitoes, ticks, and fleas, could expand (61, 62). This expansion brings with it an increased risk of disease transmission to new areas and populations.

Viral spread is also closely associated with the specific age groups it impacts, a recurring observation from past to present pandemics. Active, socially connected populations may drive rapid expansion, while severe effects on less active seniors can pressure healthcare systems. During the 1918 influenza pandemic, for example, typically influenza resistant young adults experienced high infection and mortality rates, resulting in an atypical W shaped mortality curve (51, 63, 64). The mobility and social habits of this demographic likely contributed to the rapid and widespread transmission of the virus. Additionally, the high mortality within young adults during this pandemic, which are central to the global workforce, also led to highly detrimental economic impacts. Conversely, in the 2009 H1N1 influenza pandemic, children, and teenagers were disproportionately affected, likely due to partial preexisting immunity in older populations (65). This demographic's dense social networks, particularly in school settings, played a crucial role in accelerating the spread of the virus. Schools often act as amplification settings for influenza transmission, as seen in the heightened transmission rates following school terms. The COVID-19 pandemic offers another perspective, where initially older adults (65+ years) were more severely affected (66). While their mobility might be lower, thus potentially reducing the spread, the high morbidity and mortality rates in this group led to significant healthcare burdens. However, as the pandemic progressed, the role of younger adults in transmission became more evident, especially in driving community spread (66).

Viral outbreaks and pandemics that affect humans often originate from animals. Except for smallpox, whose initial origin is lost to history (18), all pandemic-causing viruses assessed in this perspective are zoonotic viruses. They initially emerged from animal hosts before adapting to humans, an occurrence which is becoming increasingly common due to climate change and habitat encroachment (67, 68). HIV is believed to have originated from nonhuman primates (22), while influenza pandemics have emerged from avian and swine reservoirs (69). SARS-CoV, MERS-CoV, and SARS-CoV-2 are all associated with bats and intermediate hosts such as palm civets, camels, and pangolins, respectively (70–72). Therefore, it is crucial to monitor potential reservoirs of zoonoses as a key aspect of pandemic preparedness.

### Incubation period

Incubation period, or the time between infection and symptom development, varies within and across virus families. It serves as a critical factor in pandemic preparedness and management, informing policies like quarantine duration (73). Information on incubation periods may be limited early in an outbreak as contact tracing and symptom tracking take time. However, a recent study by Gussow et al. suggests that genetic information alone can predict the incubation period. Their model demonstrates that by knowing only the number of protein-coding genes and the guanine-cytosine content, the incubation time ranges can be accurately estimated for many RNA viruses, including SARS-CoV-2 (74).

Individuals infected with a virus with a short incubation period will develop symptoms rapidly, which could facilitate detection early in potential pandemic and allow for the implementation of containment measures. However, a short incubation period also means there is a smaller window for detection, which would be further exacerbated by the presence of asymptomatic spread during incubation. It is worth highlighting that a highly contagious virus with both a short incubation period and asymptomatic spread has the potential to overwhelm the healthcare system and rapidly escalate a pandemic, as exemplified by the 1918 influenza pandemic with its incredibly short incubation period of just 1 to 2 days (75). In a matter of months, it spread across the globe, putting strain on healthcare systems and leading to a surge in hospital admissions (76). Similar scenes were observed during the COVID-19 pandemic. However, unlike the 1918 influenza pandemic, the rapid spread was not due to short incubation periods. Instead, a longer incubation period of up to 14 days, combined with asymptomatic transmission, allowed SARS-CoV-2 to delay detection and containment measures (77). The presence of these asymptomatic individuals contributed significantly to the widespread transmission of SARS-CoV-2, ultimately shaping the COVID-19 pandemic.

### Symptoms

The severity of symptoms by those infected by a virus, as well a high mortality rate, can directly influence transmission rates. While counterintuitive, it is a known phenomenon that there is an inverse relationship between the mortality of a virus and its transmission potential, which directly impacts pandemic risk (78). This concept, known as the “transmission–virulence tradeoff,” suggests that highly deadly viruses, such as Ebola, often kill their hosts rapidly, thereby reducing the chances of spreading to others (79). This tradeoff implies that viruses with high lethality tend to have lower transmission rates because the severe symptoms incapacitate the host and limit their interactions with others. Additionally, severe symptoms and higher mortality will often lead to people staying home, reducing the opportunity for the virus to spread. The severity of symptoms can also influence the implementation and adherence to containment measures like school closures or travel restrictions, as was evident during the MERS outbreak, where the mortality rate was around 34% (80). On the other hand, individuals with mild to moderate symptoms often continue their daily activities, inadvertently increasing the risk of transmission through interactions with others. Take the 2009 H1N1 pandemic as an example, where typically mild symptoms led to the infection of 11 to 21% of the global population (81, 82).

Asymptomatic infections played a significant role in the spread of H1N1 and other influenza pandemics. In fact, they were a major driver of the Influenza A (H1N1) pandemics, with around 23% of cases caused by this virus type being asymptomatic (83). This type of transmission also greatly contributed to the COVID-19 pandemic, with approximately 25% of all cases up to August 2021 resulting from asymptomatic transmission (84).

A wide array of symptoms or symptoms that might be mistaken for other illnesses can pose a challenge when managing a pandemic, particularly during its initial stage. For instance, the early symptoms of HIV infection can be mild and flu-like, leading to decreased or misdiagnosis and ongoing transmission (85). During this acute phase, lasting weeks to months, the viral load is high, elevating the risk of transmission. The disease then transitions into a chronic, often asymptomatic phase. Despite lower viral loads during this period, transmission is still possible if treatment is not received. As the disease progresses to AIDS, symptoms reemerge and intensify, becoming more severe and debilitating (85). Just like during the early stage of HIV infection, symptoms of SARS-CoV-2 can often be mistaken for other conditions, such as the common cold or influenza (86). Moreover, this virus manifests a diverse array of symptoms, ranging greatly in severity and can be anything from loss of taste to severe respiratory distress and even death. This wide range of symptoms and varying severity has complicated the ability to quickly identify cases and contain the outbreak.

While the exact symptom type and severity may be uncertain at the beginning of an outbreak, scientists can use various techniques to make predictions. One such method involves in vitro tests like tissue binding assays to determine the viral tissue tropism, or its affinity for specific tissues (87). This can provide insights into which organs might be affected and therefore help predict potential symptoms. For example, SARS-CoV-2 tropism has been identified in multiple organs, primarily the respiratory system (e.g. lungs and trachea) but also in the kidneys, small intestines, pancreas, blood vessels, and other tissues (88). The diverse tropism of SARS-CoV-2 may therefore explain the diverse range of symptoms experienced by those infected with the virus. Insights can be gained from other intrinsic viral characteristics such the viral sequence data (e.g. binding protein sequence) and replication rate (32, 89). Lastly, comparison with closely related viruses can provide valuable information. If a new virus belongs to a well-studied family, this existing knowledge can be drawn upon to make educated guesses about its behavior, including symptom type and severity. Such assessments were done early into the COVID-19 pandemic (90).

## The ViTAP model for pandemic risk prediction

A key component of pandemic preparedness is the creation of a risk assessment model that can identify characteristics of future outbreaks that may escalate into pandemics. To address this, we have developed the first generation of the ViTAP model which encompasses a pandemic risk assessment framework. This framework is specifically designed to identify and evaluate the risk factors associated with potential pandemic viruses. It incorporates multiple categories that encompass inherent aspects of virus biology, transmissibility, and disease manifestation (Table 3). Each indicator within these categories has been assigned a score based on an extensive literature review, resulting in a cumulative risk profile.

**Table 3.** The ViTAP model framework.

Category	Score	Weight (%)
Ability to mutate and adapt		
Nucleic acid configuration	DNA = 1, RNA = 2	12
Virus family	Others = 1, Influenza A/Betacoronavirus = 2	5
Replication machinery	High fidelity = 1, Moderately error prone = 2, Very error prone = 3	2
Segmented genome	No = 1, Yes = 2	2
Evidence of reassortment event	No = 1, Yes = 2	2
Expected impact of mutations on virulence and/or transmissibility	Low = 1, Moderate = 2, High = 3	14
Transmissibility		
Primary mode of transmission	Vector-borne = 1, Sexual = 2, Contact (direct or indirect) = 3, Droplet = 4, Aerosol = 5	11
Prevalence of aerosol transmission	No = 1, Yes – rare = 2, Yes – frequent = 3	11
Presence of asymptomatic transmission	No = 1, Yes – rare = 2, Yes – frequent = 3	11
Human-to-human transmission	No = 1, Yes – rare = 2, Yes – frequent = 3	11
Age of impacted demographic	65 + years old = 1, 40–60 years old = 2, 0–17 years old = 3, 18–39 years old = 4	4
Incubation period		
Incubation period (± asymptomatic transmission)	>7 days (−) = 1, 1 to 4 days (−) = 2, 5 to 7 days (−) = 3, 1 to 7 days (+) = 4, > 7 days (+) = 5	5
Symptoms		
Similarity of symptoms to other diseases	Low = 1, Moderate = 2, High = 3	4
Mortality rate	Very low (<0.1%) = 1, Low (0.1 to 1%) = 2, Very high (>10%) = 3, High (5–10%) = 4, Moderate (1–5%) = 5	6

The ViTAP model was developed using the assumptions listed in Supplementary Table S1, including that the understanding of data surrounding an emerging virus evolves throughout the outbreak. Some viral characteristics, particularly incubation period and symptom type and severity, may be unknown or poorly understood. As such, a data quality score has been built into the ViTAP model that weights each viral characteristic score by the confidence in the available data (Equation 1). Determining a confidence level for each characteristic may largely be dependent on user knowledge at the time of assessment; however, factors such as data available for similar viruses, or lack thereof, may influence data confidence. For the purpose of assessing past and ongoing epidemics and pandemics, where viral characteristics are well defined, the data confidence level has been set at 100%.

**Viral Characteristic Quality Score**


(1) $$\mathrm{Quality}\;\mathrm{Score}=\overset{n}{\underset{i=1}{\sum }}\;(\mathrm{Viral}\;\mathrm{Characteristic}\;\mathrm{Scor}e_{i}\times \mathrm{Confidence}\;\mathrm{Leve}l_{i})$$

The approach was further refined by developing a weighted scoring system to quantify each of the evaluated factors. These weights, while subjective, were determined as a result of the comprehensive literature review provided above. Full justification of weights used in the ViTAP model is presented in Supplementary Table S2. To obtain a ViTAP score, each viral category score is multiplied by their respective weight, following equation:

**ViTAP score**


(2) $$\mathrm{ViTAP}\;\mathrm{Score}=\overset{n}{\underset{i=1}{\sum }}\;(\mathrm{Viral}\;\mathrm{Characteristic}\;\mathrm{Scor}e_{i}\times \mathrm{Quality}\;\mathrm{Scor}e_{i}\times \mathrm{Weigh}t_{i})$$

The viral characteristic identified to have the most significant impact on whether a virus emerges as a global threat is the expected impact of mutations on virulence and transmissibility, accounting for 13% of the total weighting system. Mutations that produce a more aggressive or easily transmissible strain can significantly increase the risk of a virus reaching pandemic levels. Such mutations can lead to a virus evading preexisting immunity within a population that was obtained from previous exposures or vaccinations. As such, the impact of mutations on virulence and transmissibility can serve as a proxy for understanding how preexisting immunity influences the course of an outbreak, a factor that cannot be known using inherent viral characteristics alone. Additionally, understanding the potential impact of mutations not only helps differentiate the pandemic potential of various viruses but also distinguishes between different strains of the same virus, such as a typical seasonal influenza A strain and a high pandemic potential influenza strain. However, the analysis of such mutations currently requires significant subject matter expertise and will require the use of additional models, such as the EVEscape model developed by Thadani et al. (91), which assesses the likelihood of a mutation escaping the immune response on the basis of the probabilities of a given mutation to maintain viral fitness and disrupt antibody binding.

Looking back at previous pandemics, it is clear that with the exception of HIV, viruses causing modern pandemics to have belonged to either the influenza A or betacoronavirus families. Two epidemics with high pandemic potential, SARS-CoV, and MERS-CoV, also belonged to the betacoronavirus category. For that reason, these families were designated as higher risk within the ViTAP model. It is important to note, however, that health agencies (e.g. WHO) have identified a number of other high-priority viral diseases, even including a yet unknown virus responsible for “Disease X,” that could be responsible for future pandemics (92). As such, the viral family characteristic was weighted at 5%. Instead, the ViTAP model places greater weight on whether a virus is a DNA or RNA virus (12%). Given the higher mutation rates observed in RNA viruses, they were scored higher than DNA viruses. This conclusion is also reflected in the WHO's list of high-priority viruses, which at present primarily includes RNA viruses, although they did include poxviruses in their 2024 update (93). Likewise, the list of National Institute of Allergy and Infectious Diseases’ (NIAID's) Biodefense Pathogens only includes one category of DNA virus, poxviruses (e.g. smallpox and Mpox), both of which are assessed in the ViTAP model below (94).

Many pathogenic viruses belong to the RNA viral category. To further narrow down RNA viruses of interest, modifiers such as the fidelity replication machinery were included and weighted at 2%. The presence of a segmented genome, weighted at 2%, was also included as a distinguishing viral characteristic as several viral families on the WHO high-priority list have this characteristic, including the Lassa virus (92, 95). Segmented viruses also have a higher risk of undergoing reassortment events (e.g. antigenic shift), and recent evidence of such events, weighted at 3% in the ViTAP model, suggests that a virus has greater potential to evade population-wide preexisting immunity and lead to a global outbreak. The low weights were assigned as a recognition that they are highly dependent on the RNA viral category and are included to distinguish between RNA viruses.

Transmissibility is another key factor in viral spread, and existing models typically use R_0_ as a primary indicator pandemic impact. However, *R*_0_ for a novel virus cannot be known at the start of an outbreak. Instead, the ViTAP model includes viral characteristics that can be used as proxies for *R*_0_, such as the primary mode of transmission, the prevalence of aerosol transmission, asymptomatic transmission, and the prevalence of human-to-human transmission are all equally weighted in the ViTAP model framework (11%). These factors all contribute to the ability of a virus to proliferate within a population. Similarly, the age of the impacted demographic also determines how rapidly a virus spreads due to the differences in social activities between the age groups. However, this characteristic may be difficult to accurately determine early in an outbreak, multiple demographics may be affected, and the immunocompromised status may affect the impact on those outside the primarily affect group; therefore, this is weighted at 4%.

Other viral characteristics, such as incubation period (5%) and type and severity of symptoms, serve as indicators for how quickly the virus will be identified in individuals and detected among a broader population. Such identification is key to implementing containment measures pharmaceutical interventions that could stop a regional outbreak from spreading globally.

Of these viral characteristics, the severity of symptoms likely has the largest impact on viral spread and a population's behavior in response to an outbreak. The full impact of a virus’ symptoms, such as long-term disability within a population, cannot be known early in an outbreak. Instead, the ViTAP model uses mortality rate (6%) as an indication of symptom severity. The scoring for mortality rate places a higher emphasis on viruses with moderate mortality (1–5%), as all modern pandemic viruses assessed in this review fell within that range. Counterintuitively, a virus with a higher mortality rate (e.g. >5%) will often limit its own transmission by killing those infected before they can spread the disease to others and will likely result in higher compliance rates with containment measures such as quarantines and mask measures (78). Accurately determining mortality rates, however, can be challenging early in an outbreak due to underreporting of asymptomatic cases and delays between infection, hospitalization, and death. Similarly, identifying the full range of symptoms caused by a virus may not be possible until numerous cases have been observed and studied. As such, the “Similarity of Symptoms to Other Diseases” characteristic is only weighted at 4%.

### ViTAP scores in the context of historical global or regional impact

**Fig. 2.**
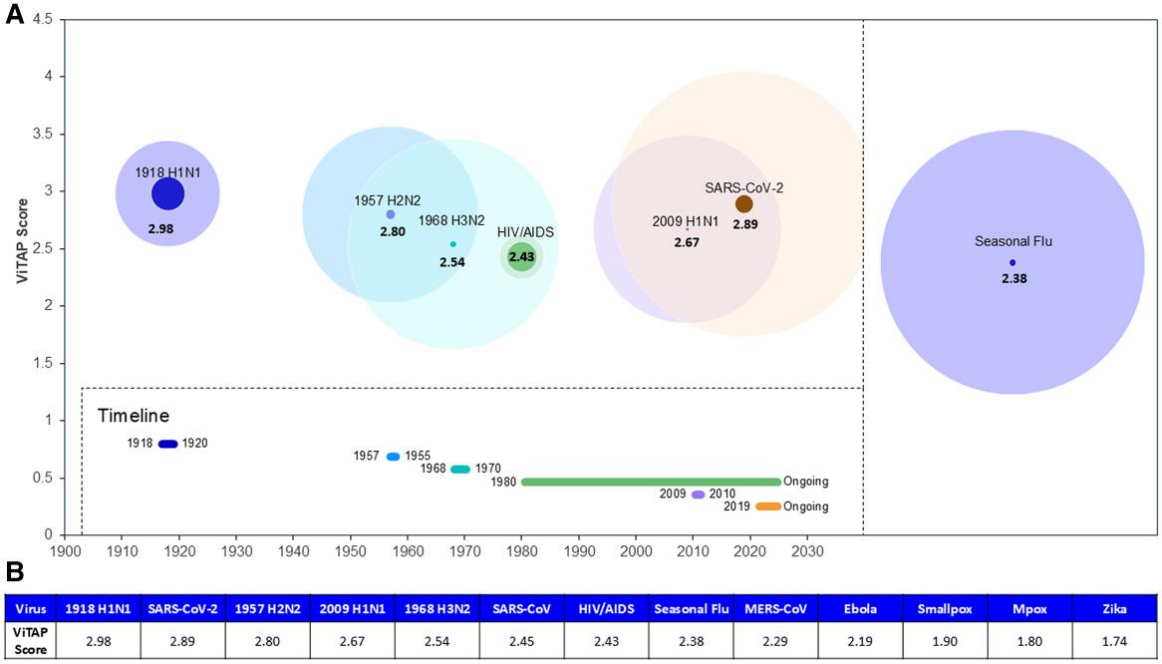
ViTAP scores in context with global impact of historical and ongoing pandemics. A) The start date and timeline for each pandemic is presented along the *x*-axis (Left). The relationship between global impact (bubble size) is compared with the ViTAP scores (*y*-axis) for each pandemic. Darker bubbles represent the number of deaths and lighter bubbles represent the number of infections attributed to each pandemic. Please note, the number of confirmed infections provided for SARS-CoV-2 and the number of actual cases is likely much higher. For comparison with pandemic viruses, the ViTAP score and global impact of an average flu season is provided (Right). B) A summary of the ViTAP scores for all viruses assessed using the pandemic risk assessment model.

The results of the ViTAP model when applied to past outbreaks (Figure 2; Supplementary Tables S2 and S3) offer valuable insights into the potential regional and global impact of pandemic and epidemic viruses (Supplementary Table S4). By considering factors like genetic makeup, transmission modes, incubation period, symptoms, and adaptability through mutation, this model empowers us to identify viruses with a higher risk profile and a greater potential to cause pandemics.

Smallpox, once a highly devastating virus, now holds the lowest ViTAP score of 1.90 of any pandemic assessed in this perspective (Supplementary Table S3). However, this is still higher than the ViTAP scores for the Zika and Mpox outbreaks. This rating, however, understates the true global impact it had, claiming an estimated 300 million lives in the 20^th^ century alone (96). Nonetheless, the ViTAP model is designed to specifically address future potential pandemics. If smallpox were to emerge today, its worldwide consequences would likely not match those of centuries past. It is well-defined and severe symptoms and slow mutation rate as a DNA virus would hinder its ability to evade vaccine efforts which, combined with relentless vaccination campaigns, lead to smallpox becoming first-ever eradicated virus in the year 1980 (97). Other factors not included in the ViTAP framework, including advancements in healthcare, improved communication, and a stockpile of smallpox vaccines, would improve our chances for swift containment and prevention, even if an epidemic were to occur.

Mpox, similar to smallpox, is a viral disease that displays characteristics akin to its eradicated counterpart. It received a ViTAP score of 1.80 (Supplementary Table S3). Both diseases belong to the same virus family, Poxviridae, and manifest similar symptoms, including fever, headache, muscle aches, backache, swollen lymph nodes, chills, and exhaustion (2). A rash then develops, often beginning on the face and then spreading to other parts of the body (2). As of August 2023, Mpox has infected approximately 93,000 people resulting in 171 confirmed deaths, globally (98). Several factors have prevented Mpox from reaching pandemic levels, one of which is its transmission mode. Mpox primarily spreads through direct contact with the blood, bodily fluids, or cutaneous or mucosal lesions of infected animals. Human-to-human transmission can also happen through large respiratory droplets during long periods of face-to-face contact (99). Additionally, those vaccinated for smallpox appear to have some immunity to Mpox, further preventing its widespread transmission (100).

When the ViTAP model is applied to past pandemic influenza strains, it becomes clear that the scores correspond with their respective global impacts. The 1918 influenza pandemic stands out as one of the most devastating and deadly pandemics in human history, receiving the highest ViTAP score of 2.98. This score is largely attributed to the “Impact of Mutations on Virulence and/or Transmissibility” and the “Mortality Rate”. The mutations that occurred allowed for such a rapid spread throughout society, infecting humans with exceptional virulence. Additionally, scientists at that time did not have the capabilities to develop a vaccine against influenza, limiting mitigation to strategies such as social distancing and quarantine. This, combined with an already high mortality rate, particularly among young adults, resulted in an estimated death toll of between 20 and 50 million people worldwide (101).

In comparison, the 1957 H2N2 pandemic, the 1968 H3N2 pandemic, and the 2009 H1N1 pandemic registered scores of 2.80, 2.54, and 2.67 respectively in terms of their impacts on global mortality. Despite causing fewer deaths than the 1918 strain, each of these pandemics still resulted in significant loss of life, with the 1957 and 1968 pandemics estimated to be responsible for 1–2 million deaths each, and the 2009 pandemic believed to have caused between 151,700 and 575,400 fatalities worldwide. In all these cases, the virus's ability to mutate and the resulting changes in its virulence and transmissibility played pivotal roles in their global dissemination.

In conjunction with subject matter expertise, the proposed risk assessment model can be used to indicate high-risk influenza strains. For example, an average influenza A season received a ViTAP score of 2.38 Supplementary Table S3 due to the lower mortality rate and a reduced impact of mutation on both virulence and transmissibility when compared with pandemic strains. Thus, understanding the nuances of mutations and their potential to cause pandemics is essential for managing future influenza outbreaks.

Of all the betacoronaviruses assessed in this perspective, SARS-CoV-2 has the highest ViTAP score of 2.89 and the most significant global impact in the form of the COVID-19 pandemic. This virus has impacted almost every country, with over 700 million confirmed cases and millions of lives lost worldwide. Its elevated risk score can be attributed to its novel virulence factors, efficient respiratory transmission, and a wide range of symptoms, spanning from asymptomatic to severe cases. Additionally, while coronaviruses, including SARS-CoV-2, possess a unique RNA-dependent RNA polymerase (RdRp) proofreading capability that reduces replication errors (32), their fidelity is still not as high as that of DNA viruses, resulting in a replication machinery score of 2. The virus has a demonstrated ability mutate and adapt, leading to the emergence of new variants that may further impact global health (102).

Compared with SARS-CoV-2, the global impacts of other betacoronavirus outbreaks were moderate. SARS-CoV emerged in 2002 and infected approximately 8,000 people, resulting in 774 lives lost. This virus spread efficiently through respiratory droplets but had a very high mortality rate (11%), earning it a ViTAP score of 2.45 (103). This high mortality rate has been attributed to the swift and assertive response from public health authorities in outbreak hotspots, such as Singapore and Toronto, and the population's compliance with these measures, leading to the containment of the virus (104). Such strategies were also employed during the COVID-19 pandemic; however, they were less effective in part due to high prevalence of asymptomatic transmission which contributed to greater spread (105). Like SARS-CoV, MERS-CoV has had a relatively moderate global impact. During the outbreak, the Middle East was hit hardest, with sporadic cases appearing in Europe, Asia, and the United States. Approximately 2,500 people have been infected by MERS-CoV, resulting in 866 deaths (80). The comparatively lower risk score of 2.29 is attributed to its limited human-to-human transmission capabilities.

The other two outbreaks assessed, Ebola and Zika, received ViTAP scores of 2.19 and 1.74, respectively, in this pandemic risk assessment framework. The Ebola virus has caused several outbreaks, most notably the West Africa Ebola epidemic from 2014 to 2016, resulting in over 11,000 fatalities (106). Its high mortality rate, along with its restricted human-to-human transmission and geographical containment, have kept it from becoming a pandemic (106). On the other hand, the Zika virus has had a less immediate impact on mortality, with only 51 confirmed deaths between 2015 and 2016 (107). However, the repercussions of Zika extend beyond this measurement. It can induce severe birth defects in babies of infected pregnant women, including microcephaly, where babies are born with considerably smaller heads, leading to developmental challenges (108). The 2015 to 2016 Zika epidemic, which spread swiftly across the Americas, saw a significant surge in such cases, creating a significant public health concern.

While the impact on mortality of these outbreaks was limited, they still had significant economic implications for the impacted regions, encompassing direct healthcare costs for diagnosis and treatment, productivity loss, and negative impacts on tourism and trade. For example, the financial strain of the Zika outbreak in Latin America and the Caribbean alone is estimated to reach billions of dollars, incorporating both direct healthcare costs and the wider socioeconomic impact (109). Thus, while Ebola and Zika garner relatively low ViTAP scores, mainly due to aspects like their primary transmission modes and geographical distribution, it is important to remember that a virus's overall impact is not solely determined by its pandemic potential. The direct and indirect health effects, along with broader societal and economic repercussions, are key factors in comprehensive public health preparedness and response strategies.

**Fig. 3.**
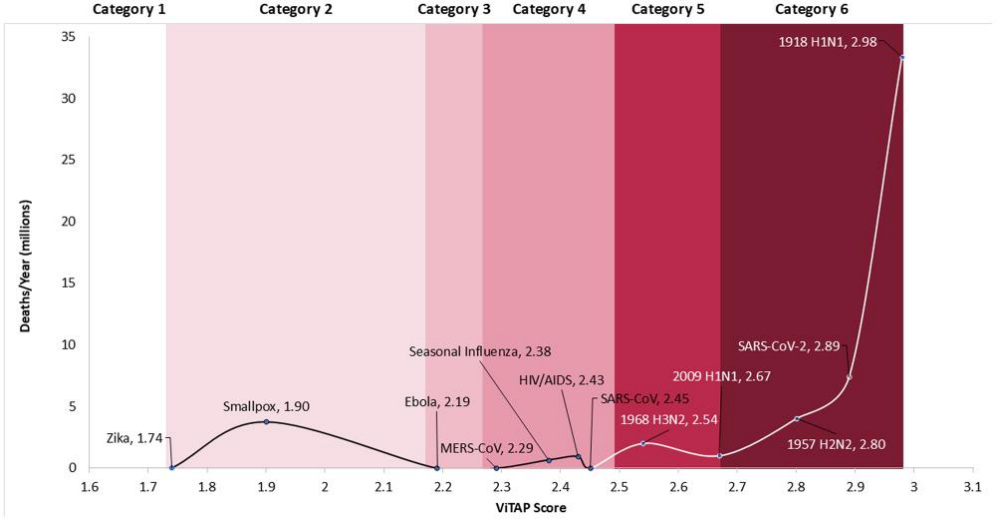
Pandemic risk categorization system using the ViTAP model. Illustrative example of a risk assessment categorization system for novel or outbreak viruses using ViTAP scores of past pandemics correlated with deaths per year over the course of the pandemic.

Such a tool would also need a way of classifying viral risk. Here, we propose a classification system analogous to the system used to categorize hurricane risk. Such a pandemic risk categorization system would use ViTAP scores for historical outbreaks compared to a measurable metric of human impact (e.g. mortality rates and Disability-Adjusted Life Years [DALYs]). The proposed stratification, ranging from Category 1 representing viruses with minimal impact, to Category 6 denoting pathogens with high risk for severe global consequences, could be used to assess the potential risk of an emerging virus and provide guidance to researchers, pharmaceutical manufactures, and public health officials on which viruses to focus resources on. It could also be used to identify viruses that are not likely to escalate to a global outbreak, therefore allowing the stakeholders identified above to redirect resources elsewhere. An illustrative example of the proposed risk category system, using mortality as a metric for global impact, is presented in Figure 3. However, additional validation and the incorporation of other determinates of global impact (e.g. DALYs) are needed before this can be used as a determinative tool.

To demonstrate the utility of the ViTAP model and the proposed risk categorization system in an early pandemic, or prepandemic situation, we used literature from before the declaration of the pandemic in March 2020 to determine how SARS-CoV-2 would have ranked based on data available (110–115). The full comparison can be found in Supplementary Table S2. The main differences were observed in its ability to mutate and adapt and its transmissibility factors. Studies on impact of mutations of spike D614G on infectivity, for example, were not published until June 2020, long after the virus had already swept the globe (116). Based on data from other coronaviruses, the fidelity of SARS-CoV-2 replication machinery early in the pandemic was also believed to be high due to the proofreading capacity of the nonstructural protein 14 (NSP14) exonuclease, which corrects errors made by the RNA-dependent RNA polymerase (RdRp) (117). However, as more detailed studies emerged, it became evident that while SARS-CoV-2's RdRp does exhibit relatively high fidelity for an RNA virus, this proofreading mechanism allowed enough mutations to accumulate, facilitating the virus's adaptability and evolution, contributing to the emergence of more transmissible and potentially more infectious variants (118). The presence of both asymptomatic and aerosol transmission were also not fully established until much later in the pandemic (112, 115). Using this information in the ViTAP model, we arrive at a score of 2.45, placing it in the risk Category 4. Despite the lower score, the ViTAP model still shows that SARS-CoV-2 early in the outbreak had a significant potential for global impact, reflecting the need for rapid response strategies. This further demonstrates the utility of the ViTAP model in providing critical early warnings to prevent the escalation of emerging viral threats into global pandemics.

### Limitations of the ViTAP model

The ViTAP model, in its current form, serves as a novel conceptual framework for assessing the pandemic potential of emerging pathogens based on their intrinsic viral characteristics. However, as with any new approach, there are inherent limitations that must be acknowledged and addressed through further research and model refinement.

One of the main challenges in developing the ViTAP model is the complex interplay between symptoms, transmissibility, and mortality. The current model does not fully capture these interactions, as incorporating such dynamics would require advanced modeling techniques and extensive datasets. The virulence-transmissibility tradeoff, a well-established concept in epidemiology, highlights the inverse relationship between disease severity and transmission potential. However, the exact nature of this relationship varies significantly depending on the specific pathogen, host factors, and environmental conditions. Symptoms play a crucial role in this tradeoff, influencing both disease severity and the ability to detect and isolate infected individuals. To fully integrate these complex dynamics into the ViTAP model, further research, and collaboration across multiple disciplines will be necessary.

Another limitation of the ViTAP model is the subjectivity of the scoring system, which relies on expert opinion and qualitative assessments of viral characteristics. Although we have provided justifications for the weights and scores assigned to each category (Supplementary Table S5), we recognize that there is room for refinement and validation through more rigorous quantitative analysis. Comparing the ViTAP model's performance against existing pandemic potential frameworks using historical outbreak data could help identify areas for improvement and better calibrate the scoring system.

Furthermore, the ViTAP model does not fully account for the interdependent nature of many viral characteristics. While we have attempted to provide modifiers for major drivers of pandemic risk (e.g. replication machinery for nucleic acid configuration), the model does not directly account for factors such as the interdependency between modes of transmission and severity of disease. Modeling the impact of these interdependencies on pandemic risk is complex and beyond the scope of this review.

It is important to note that the ViTAP model is intended as a conceptual starting point rather than a definitive tool for pandemic risk assessment. The primary goal of this perspective is to introduce a novel approach that incorporates intrinsic viral characteristics and demonstrates its potential utility in guiding pandemic preparedness efforts. As Katz and Sridhar (119) propose in their decision-making tree for policy responses to emerging pathogens, flexibility in policy response based on the characteristics of the pathogen and the tools available is crucial. The ViTAP model aims to provide a foundation for such a flexible approach by identifying key viral characteristics that influence pandemic potential. However, to fully grasp the potential for a pandemic, the use of this model must be complemented by other analytical tools and data, as well as external factors such as location of outbreak, population density, and presence of existing interventions.

Despite these limitations, we believe that the ViTAP model represents an important step forward in our understanding of pandemic risk assessment. By focusing on intrinsic viral characteristics rather than relying solely on epidemiological data that may be unavailable in the early stages of an outbreak, the ViTAP model offers a complementary approach to existing frameworks. As we continue to refine and validate this model through further research, we hope that it will serve as a valuable tool for policymakers, public health officials, and researchers in their efforts to prepare for and mitigate the impact of future pandemics.

## Outlook

The COVID-19 pandemic has highlighted the urgent need for proactive strategies to strengthen our preparedness for future outbreaks. The increasing risk of pandemics, driven by factors such as global travel, urbanization, and climate change, calls for a shift toward predictive and preventative approaches. To effectively prepare for future pandemics, we must learn from the past and understand the potential threats posed by emerging pathogens.

The ViTAP model, presented in this perspective, offers a novel approach to pandemic risk assessment by focusing on the intrinsic biological characteristics of viruses. By integrating lessons learned from past pandemics, the ViTAP model provides a framework for identifying high-risk viruses and estimating their potential impact. By embracing innovative approaches like the ViTAP model and fostering collaboration across disciplines, we can pave the way for a more resilient global health infrastructure. Through proactive preparation and swift response, we can mitigate the impacts of future pandemics and protect the health and well-being of populations worldwide.

## Supplementary Material

pgae558_Supplementary_Data

## Data Availability

All data are included in the manuscript and/or supporting information.
