# Supplementary material for: In silico prediction of pathogen's pandemic potential using the viral trait assessment for pandemics (ViTAP) model
Source: PNAS Nexus. 2024 Dec 10;3(12):pgae558. doi: 10.1093/pnasnexus/pgae558 (PMC11658415; doi:10.1093/pnasnexus/pgae558)
Supplement: pgae558_Supplementary_Data [file pgae558_supplementary_data.docx]

**Supplementary Information**

**Supplementary Table 1. Assumptions used in the development of the ViTAP model**

| **Assumptions** | |
| --- | --- |
| No Interaction Effects | The model assumes that each category contributes independently to pandemic risk, without interactions, The interplay between factors might modify the overall risk. |
| Historical Relevance | The framework is heavily based on the limited dataset of pandemic-causing viruses of the past approximately 100 years. This assumes that future pandemics will have characteristics like those of the past. |
| Binary or Ternary Categories | Several criteria are divided into yes/no or low/moderate/high classifications. This simplification might overlook nuances. |
| Weight Justifications | The weights given to each category are estimates based on perceived importance. They might not capture the actual contribution of each factor to pandemic risk. |
| Generalizability | The model assumes that its framework is universally applicable across different regions and populations. Different areas may have unique factors that affect pandemic risk. |
| Non-Consideration of External Factors | The model focuses primarily on the biological and transmission characteristics of viruses. External factors like healthcare infrastructure, public health measures, existing interventions, societal behavior, and global connectivity are not considered but can significantly influence pandemic potential. |
| Evolving Knowledge | The assumptions and categorizations are based on the current understanding of viruses and pandemics. As knowledge evolves, some of these assumptions might become outdated. |
| No Account of Co-Infections | The framework does not consider the scenario where multiple pathogens circulate simultaneously, which could influence the dynamics of a potential pandemic. |
| Severity Measures | The model categorizes symptom severity primarily based on mortality rate, which might not capture the full range of morbidity caused by a disease. |
| User Knowledge | The model assumes subject matter expertise will be used in scoring viral categories. |

**Supplementary Table 2. ViTAP Scores for Historical Pandemic Viruses**

| **Category** | **1918  H1N1** | **1957  H2N2** | **1968  H3N2** | **2009  H1N1** | **Smallpox** | **HIV-1/ AIDS** | **SARS-CoV-2** | **SARS-CoV-2  (Pre-pand.*)** |
| --- | --- | --- | --- | --- | --- | --- | --- | --- |
| Ability to Mutate and Adapt | | | | | | | | |
| Nucleic Acid Configuration | 2 | 2 | 2 | 2 | 1 | 2 | 2 | 2 |
| Virus Family | 2 | 2 | 2 | 2 | 1 | 1 | 2 | 2 |
| Replication Machinery | 2 | 2 | 2 | 2 | 1 | 3 | 2 | 2 |
| Segmented Genome | 2 | 2 | 2 | 2 | 1 | 1 | 1 | 1 |
| Evidence of Reassortment Event | 2 | 2 | 2 | 2 | 1 | 1 | 1 | 1 |
| Expected Impact of Mutations on Virulence and/or Transmissibility | 3 | 2 | 2 | 2 | 1 | 3 | 2 | 1.5 |
| Modes of Transmission | | | | | | | | |
| Primary Mode of Transmission | 4 | 4 | 4 | 4 | 4 | 2 | 4 | 4 |
| Prevalence of Aerosol Transmission | 3 | 3 | 3 | 3 | 2 | 1 | 3 | 1 |
| Presence of Asymptomatic Transmission | 2 | 2 | 2 | 3 | 1 | 3 | 3 | 2 |
| Human-to-Human Transmission | 3 | 3 | 3 | 3 | 3 | 3 | 2 | 3 |
| Incubation Period | | | | | | | | |
| Incubation Period (± asymptomatic transmission) | 4 | 4 | 4 | 4 | 1 | 5 | 4 | 4 |
| Symptoms | | | | | | | | |
| Similarity of Symptoms to Other Diseases | 3 | 3 | 3 | 3 | 2 | 1 | 2 | 2 |
| Mortality Rate | 5 | 3 | 3 | 3 | 2 | 1 | 5 | 5 |
| **ViTAP Score** | **2.98** | **2.80** | **2.54** | **2.67** | **1.90** | **2.43** | **2.89** | **2.45** |
| *Information used to assess pre-pandemic SARS-CoV-2 was published between December 2019 and March 2020. | | | | | | | | |

**Supplementary Table 3. ViTAP Scores for Historical Non-pandemic Viruses**

| **Category** | **SARS-CoV** | **MERS-CoV** | **Seasonal Influenza** | **Ebola** | **Zika** | **Mpox** |
| --- | --- | --- | --- | --- | --- | --- |
| Ability to Mutate and Adapt | | | | | | |
| Nucleic Acid Configuration | 2 | 2 | 2 | 2 | 2 | 1 |
| Virus Family | 2 | 2 | 2 | 1 | 1 | 1 |
| Replication Machinery | 2 | 2 | 2 | 3 | 2 | 1 |
| Segmented Genome | 1 | 1 | 2 | 1 | 1 | 1 |
| Evidence of Reassortment Event | 1 | 1 | 2 | 1 | 1 | 1 |
| Expected Impact of Mutations on Virulence and/or Transmissibility | 3 | 3 | 1 | 3 | 2 | 1 |
| Modes of Transmission | | | | | | |
| Primary Mode of Transmission | 4 | 4 | 4 | 4 | 1 | 3 |
| Prevalence of Aerosol Transmission | 3 | 2 | 3 | 1 | 1 | 2 |
| Presence of Asymptomatic Transmission | 1 | 1 | 2 | 1 | 2 | 1 |
| Human-to-Human Transmission | 3 | 3 | 3 | 3 | 3 | 2 |
| Incubation Period | | | | | | |
| Incubation Period (± asymptomatic transmission) | 2 | 1 | 4 | 1 | 2 | 1 |
| Symptoms | | | | | | |
| Similarity of Symptoms to Other Diseases | 1 | 1 | 3 | 1 | 1 | 2 |
| Mortality Rate | 1 | 1 | 2 | 3 | 1 | 5 |
| **ViTAP Score** | **2.45** | **2.29** | **2.38** | **2.19** | **1.74** | **1.80** |

**Supplementary Table 4. Global impact of pandemic and non-pandemic viruses compared with their ViTAP score**

| **Virus** | **ViTAP Score** | **Cases** | **Death** | **References** |
| --- | --- | --- | --- | --- |
| 1918 H1N1 | 2.97 | 500,000,000 | 50,000,000 | 1,2 |
| SARS-CoV-2 | 2.83 | 3,160,000,000^a^ | 14,830,000^b^ | 3,4 |
| 1957 H2N2 | 2.80 | 1,400,000,000^c^ | 4,000,000 | 5-7 |
| 2009 H1N1 | 2.67 | 1,600,000,000^d^ | 575,000 | 5,8,9 |
| 1968 H3N2 | 2.54 | 2,000,000,000 | 2,000,000 | 5,10 |
| SARS-CoV | 2.49 | 8,096 | 774 | 11 |
| HIV/AIDS | 2.41 | 85,600,000 | 40,400,000 | 12 |
| Seasonal Influenza | 2.38 | 1,000,000,000 | 650,000 | 13 |
| MERS-CoV | 2.27 | 2,605^e^ | 937^e^ | 14 |
| Ebola | 2.17 | 28,652 | 11,325 | 15 |
| Smallpox | 1.90 | Unknown | 300,000,000^f^ | 16 |
| Mpox | 1.80 | 92,783 | 171 | 17 |
| Zika | 1.73 | >707,133^g^ | 51^h^ | 18,19 |
| a. Estimate cases based on global population in 2021 and assuming 50% of percent of global population infected by November 14, 2021.  b. Estimate deaths based on excess mortality.  c. Estimate cases based on global population in 1957 and estimate 50% of percent of global population infected. d. Estimate cases based on global population in 2009 and estimate 24% of percent of global population infected. e. Confirmed cases and deaths as of October 25, 2023.  f. Estimate from the 20^th^ century only.  g. Data from May 15, 2015, to December 15, 2016.  h. Confirmed deaths between 2015 to 2016. | | | | |

**Supplementary Table 5. Justification for weights of viral categories used in the ViTAP model**

| **Category** | **Weight (%)** | **Justification** |
| --- | --- | --- |
| Expected Impact of Mutations on Virulence and/or Transmissibility | 14% | This assessment is key to differentiating the pandemic risk of different strains within the same viral family. |
| Nucleic Acid Configuration | 12% | Pandemics of the past approx. 100 years have been RNA viruses, making this a strong predictor of future pandemics. |
| Primary Mode of Transmission | 11% | These factors all greatly contribute to a viruses’ ability to spread and serve as an early indication of R_0_, which may not be known early in an outbreak. |
| Human-to-Human Transmission | 11% |  |
| Presence of Asymptomatic Transmission | 11% |  |
| Prevalence of Aerosol Transmission | 11% | Aerosol transmission can greatly enhance virus spread. However, the prevalence may also not be evident at the start of an outbreak. |
| Mortality Rate | 6% | Mortality rate also contributes to viral spread and is a key determinant of the global impact. However, mortality rate, as a lagging indicator, may not be known early in an outbreak. |
| Virus Family | 5% | The virus families Influenza A and betacoronaviruses have shown high pandemic potential in the past. |
| Incubation Period | 5% | Incubation period also contributes viral spread; however, data may be limited early in an outbreak. May be able to estimate using GC content. |
| Age of Impacted Demographics | 4% | Age of impacted demographics has a direct impact on the ability of a virus to spread within a population due to differences in activity levels and sociological behaviors. |
| Similarity of Symptoms to Other Diseases | 4% | Similar symptoms to common illnesses can hinder early detection and response, contributing to spread. However, the full spectrum of symptoms may not be evident at the start of an outbreak. |
| Evidence of Reassortment Events | 2% | Reassortment events can give rise to novel strains with high pandemic potential. However, non-pandemic strains can also result from such events. |
| Replication Machinery | 2% | These factors contribute to a viruses’ ability to mutate and adapt. However, they are both dependent on nucleic acid configuration and virus family. |
| Segmented Genome | 2% |  |

**References**

1 Taubenberger, J. K., Morens, D. M. 1918 Influenza: the mother of all pandemics. *Revista Biomedica* **17**, 69-79 (2006).

2 Johnson, N. P., Mueller, J. Updating the accounts: global mortality of the 1918-1920” Spanish” influenza pandemic. *Bull. Hist. Med.*, 105-115 (2002).

3 Barber, R. M. *et al.* Estimating global, regional, and national daily and cumulative infections with SARS-CoV-2 through Nov 14, 2021: a statistical analysis. *Lancet* **399**, 2351-2380 (2022).

4 Msemburi, W. *et al.* The WHO estimates of excess mortality associated with the COVID-19 pandemic. *Nature* **613**, 130-137 (2023).

5 Saunders-Hastings, P. R. & Krewski, D. Reviewing the History of Pandemic Influenza: Understanding Patterns of Emergence and Transmission. *Pathogens* **5**, 66 (2016).

6 World Meters. *World Population by Year*, https://www.worldometers.info/world-population/world-population-by-year/ (2023).

7 Viboud, C. *et al.* Global Mortality Impact of the 1957–1959 Influenza Pandemic. *J. Infect. Dis.* **213**, 738-745 (2016).

8 Van Kerkhove, M. D., Hirve, S., Koukounari, A., Mounts, A. W. Estimating age-specific cumulative incidence for the 2009 influenza pandemic: a meta-analysis of A(H1N1)pdm09 serological studies from 19 countries. *Influenza Other Respir. Viruses* **7**, 872-886 (2013).

9 CDC. *First Global Estimates of 2009 H1N1 Pandemic Mortality Released by CDC-Led Collaboration*, https://www.cdc.gov/flu/spotlights/pandemic-global-estimates.htm#:~:text=The%20study%2C%20co%2Dauthored%20by,first%20year%20the%20virus%20circulated. (2012).

10 Jinjarak, Y., Noy, I., Ta, Q. Pandemics and Economic Growth: Evidence from the 1968 H3N2 Influenza. *Economics of Disasters and Climate Change* **6**, 73-93 (2022).

11 WHO. *Summary of probable SARS cases with onset of illness from 1 November 2002 to 31 July 2003*, https://www.who.int/publications/m/item/summary-of-probable-sars-cases-with-onset-of-illness-from-1-november-2002-to-31-july-2003 (2015).

12 WHO. *HIV*, https://www.who.int/data/gho/data/themes/hiv-aids#:~:text=Since%20the%20beginning%20of%20the,at%20the%20end%20of%202022. (2023).

13 WHO. *Influenza (Seasonal)*, https://www.who.int/news-room/fact-sheets/detail/influenza-(seasonal) (2023).

14 WHO, *Middle East respiratory syndrome coronavirus (MERS-CoV)*, https://www.who.int/health-topics/middle-east-respiratory-syndrome-coronavirus-mers#tab=tab_1 (2024)

15 CDC. *2014-2016 Ebola Outbreak in West Africa*, https://www.cdc.gov/vhf/ebola/history/2014-2016-outbreak/index.html#:~:text=On%20March%2029%2C%202016%2C%20the,%2C%20Liberia%2C%20and%20Sierra%20Leone. (2019).

16 Chaib, F. *WHO commemorates the 40th anniversary of smallpox eradication*, https://www.who.int/news/item/13-12-2019-who-commemorates-the-40th-anniversary-of-smallpox-eradication#:~:text=Until%20it%20was%20wiped%20out,contained%20in%20Somalia%20in%201977. (2019).

17 WHO. *2022-23 Mpox (Monkeypox) Outbreak: Global Trends*, https://worldhealthorg.shinyapps.io/mpx_global/ (2023).

18 Cardona-Ospina, J. A. *et al.* Fatal Zika virus infection in the Americas: A systematic review. *Int. J. Infect. Dis.* **88**, 49-59 (2019).

19 Ikejezie, J. *et al.* Zika Virus Transmission - Region of the Americas, May 15, 2015-December 15, 2016. *MMWR Morb. Mortal Wkly Rep.* **66**, 329-334 (2017).
